# Berberine and Berberine-Derived Compounds as Promising Weapons Against *Helicobacter pylori*: A Narrative Review

**DOI:** 10.3390/ph19081279

**Published:** 2026-08-13

**Authors:** Szymon Viscardi, Anna Duda-Madej, Paweł Krzyżek

**Affiliations:** 1 Department of Pharmacology, Faculty of Medicine, Wroclaw Medical University, Mikulicza-Radeckiego 2, 50-345 Wrocław, Poland; szymon.viscardi@student.umw.edu.pl; 2 Department of Microbiology, Faculty of Medicine, Wroclaw Medical University, Chałubińskiego 4, 50-368 Wrocław, Poland; pawel.krzyzek@umw.edu.pl

**Keywords:** berberine, berberine derivatives, biofilm formation, cytotoxins, *Helicobacter pylori*, nanoformulations, outer membrane vesicles, urease

## Abstract

*Helicobacter pylori* is one of the most common bacterial pathogens in humans and the primary etiological agent of chronic gastritis, peptic ulcer disease, and gastric cancer. Its ability to establish persistent gastric colonization relies on multiple virulence factors, including adhesins, urease, cytotoxins, motility, outer membrane vesicles, and biofilm formation, which collectively promote bacterial survival, chronic inflammation, and treatment failure. The increasing prevalence of antibiotic-resistant *H. pylori* strains has intensified the search for therapeutic strategies targeting both bacterial viability and virulence. Berberine (BBR), a natural isoquinoline alkaloid, has emerged as a promising candidate because of its antibacterial, anti-inflammatory, and antioxidant properties. Increasing evidence derived from native berberine, its derivatives, and berberine-based formulations indicates multifaceted anti-*H. pylori* activity, including direct antibacterial effects, inhibition of virulence determinants, and modulation of host inflammatory responses. This review summarizes current knowledge on the epidemiology and pathogenic mechanisms of *H. pylori* and provides a comprehensive overview of the available evidence regarding the anti-*H. pylori* pharmacological profile of BBR-based compounds. Particular attention is given to their effects on bacterial adhesion, motility, urease activity, efflux pump function, biofilm formation, and host inflammatory signaling pathways. The review also discusses findings from preclinical and clinical studies supporting BBR-based strategies as adjuncts to conventional eradication therapies. In addition, recent advances in nanotechnology-based drug delivery systems designed to overcome the poor oral bioavailability of BBR and improve its therapeutic efficacy against *H. pylori* are highlighted.

## 1. *Helicobacter pylori* Infections

### 1.1. Epidemiology

*Helicobacter pylori* infection is one of the most common chronic bacterial diseases in humans and represents a significant public health problem worldwide. It accounts for a substantial portion of the global burden of gastrointestinal diseases, including peptic ulcer disease and gastric cancer, affecting patients’ quality of life, healthcare expenditures, and work productivity [1]. Recent meta-analyses estimate that *H. pylori* infection affects approximately 43% of the world’s population. Although its frequency has shown a declining trend, it remains at a relatively high level [2,3]. Notably, this prevalence is associated with geographical differences, socioeconomic development, sanitary conditions, and access to healthcare [3].

Beyond its high incidence, one of the major clinical challenges associated with *H. pylori* infection is its chronic nature. Long-term colonization of the gastric mucosa contributes to prolonged host exposure to bacterial virulence factors and persistent immune stimulation. The resulting chronic inflammatory response promotes the establishment of a dynamic equilibrium between the microorganism and the host immune system, allowing *H. pylori* to survive despite an active immune response [4,5]. This can lead to the initiation of a sequence of pathological changes known as the Correa cascade, which includes chronic active gastritis, glandular atrophy, intestinal metaplasia, dysplasia, and ultimately gastric cancer [6,7]. This process can take several years or even decades, thereby providing an opportunity for therapeutic intervention at earlier stages. Consequently, *H. pylori* has been classified as a Group 1 carcinogen by the International Agency for Research on Cancer [8]. This has been reinforced by recent studies demonstrating that *H. pylori* infection is currently the most important known risk factor for the development of neoplastic lesions and is associated with cases of non-cardia gastric cancer worldwide [1].

### 1.2. Virulence Determinants Involved in Helicobacter pylori Colonization, Persistence, and Disease Progression

#### 1.2.1. Clinical Significance of *Helicobacter pylori*

The development of *H. pylori* infection and its clinical consequences result from a complex interaction between bacterial properties, host immune response, and environmental factors. A key role in this process is played by virulence factors, which enable these bacteria to survive in the acidic environment of the stomach, effectively colonize the mucosa, and modulate the host inflammatory response. Individual virulence mechanisms do not act independently but form a functionally linked system involving bacterial motility, adhesion to epithelial cells, neutralization of the acidic environment, and induction of cellular damage [5,9,10,11]. 

The first stage of infection is the migration of bacteria through the mucus layer covering the surface of the stomach, which is primarily caused by the spiral shape of these bacteria and the presence of polar flagella. This allows them to reach a niche with more favorable pH conditions, located in the immediate vicinity of the gastric epithelium [12,13]. Subsequently, outer membrane proteins (OMPs) participate in the stable binding of bacteria to host cell receptors, which is essential for maintaining chronic colonization [14,15]. Once bound to the cell surface, *H. pylori* can effectively utilize further virulence mechanisms responsible for the transport of toxic proteins to epithelial cells and the activation of inflammatory responses [9,16]. Long-term activation of these mechanisms leads to the maintenance of chronic inflammation, which is the basis for the development of precancerous and neoplastic changes in the stomach [9,11,17].

#### 1.2.2. Flagella and Chemotaxis as Determinants of Gastric Tropism

The ability to actively migrate through the mucus layer covering the gastric epithelium is the first prerequisite for successful colonization of the stomach by *H. pylori*. This mechanism depends on the presence of 4–6 polar flagella, the main structural elements of which are the flagellins FlaA and FlaB. FlaA is primarily responsible for the formation of the outer part of the filament, while FlaB is located closer to the cell body and participates in generating the appropriate stiffness and efficiency of movement [18,19,20]. The MotA-MotB complex located in the cytoplasmic membrane is responsible for generating the drive, which uses a proton gradient (PMF, proton motive force) to drive the rotation of the basal body of the flagellum [21]. Disturbance of *motA* or *motB* gene expression leads to loss of bacterial motility and a significant limitation of its colonization capacity, which confirms the key role of this mechanism in the pathogenesis of infection [22,23]. The motility of *H. pylori* is further controlled by a chemotaxis system including methyl-accepting chemotaxis proteins (MCPs) and CheA, CheY, and CheW proteins. This system allows bacteria to detect chemical gradients and migrate towards higher pH areas located near the surface of gastric epithelial cells [24,25,26]. Mutations in genes encoding elements of the flagella or chemotaxis lead to a significant reduction in colonization, confirming that motility is one of the first and necessary stages of infection [26]. In addition to their ability to move, flagella can also influence the interaction of bacteria with the host’s immune system, presenting lesser immunogenicity than analogous structures of many other Gram-negative bacteria, which limits recognition by innate immune receptors and promotes the chronic nature of the infection [26,27].

#### 1.2.3. Adhesins Promote Stable Colonization of Gastric Epithelial Cells

**Figure 1:**
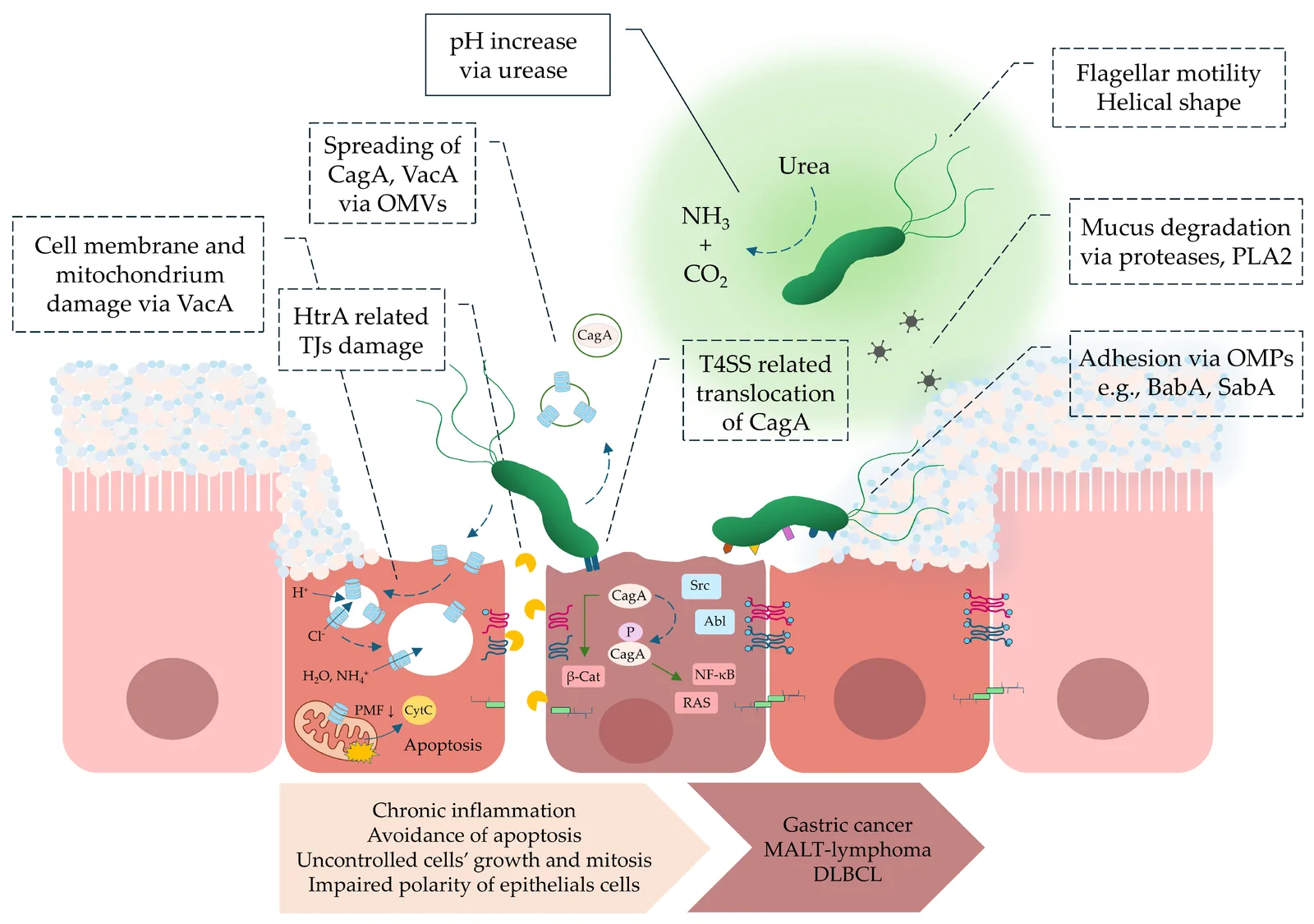
*H. pylori* pathogenesis with special regard to virulence factors crucial for stomach colonization.

Once the epithelial surface is reached, the bacterium must maintain permanent contact with the host cells. This process is mainly controlled by outer membrane proteins belonging to the Hop family (*Helicobacter* outer membrane proteins). Of the several dozen described proteins of this family, BabA, SabA, HopQ and AlpA/AlpB have the best-known pathogenetic significance [15,28,29,30]. BabA (blood group antigen-binding adhesin) is one of the most important adhesins of *H. pylori*. This protein binds the Lewis b antigen present on the surface of gastric epithelial cells and erythrocytes. This interaction increases the strength of bacterial adhesion to the mucosa and is particularly frequently observed in strains characterized by a greater ability to induce precancerous changes [31,32]. SabA (sialic acid-binding adhesin) exhibits distinct functional properties because it recognizes sialic acid-containing structures, the expression of which increases during the chronic inflammatory process. This means that SabA allows the bacterium to adapt to the changing host environment and maintain colonization even in the later stages of infection [33,34]. Variability in the expression of individual OMPs allows *H. pylori* to evade the immune response and hinders the effective control of the bacteria by the immune system [29]. HopQ is also an important element in the interaction of *H. pylori* with host cells. This adhesin binds CEACAM (carcinoembryonic antigen-related cell adhesion molecules) receptors and determines the efficient transmission of cytotoxic proteins [35]. In turn, AlpA and AlpB proteins participate in mucosal colonization and induction of the inflammatory response by activating TLR2-dependent pathways [36]. Thus, adhesins not only function as a mechanical "anchoring" of bacteria in the stomach. Their presence determines the possibility of effective action of inflammation-inducing virulence factors, which require close contact of these bacteria with the host cell. The summary of *H. pylori* virulence and pathogenesis is presented in Figure 1.

#### 1.2.4. Outer Membrane Vesicles (OMVs) Support *Helicobacter pylori* Against the Immune System

OMVs, which are physiologically released structures containing outer membrane lipids, lipopolysaccharide, and virulence proteins (including adhesins, proteases, and toxins), are also an important element in the pathogenesis of *H. pylori* [37]. OMVs are taken up by gastric epithelial cells through endocytosis, enabling the transport of bacterial effector factors into host cells. In this way, they participate in the modulation of the immune response, the induction of apoptosis of immune cells, and the intensification of chronic inflammation by regulating signaling pathways related to signaling pathways such as NF-κB [38,39,40]. Interestingly, OMVs may protect *H. pylori* from oxidative stress and support bacterial survival in the gastric environment, providing an additional mechanism promoting chronic colonization and progression of pathological changes [41]. Furthermore, exposure of *H. pylori* to antibiotics can alter both the production of OMVs and their cytotoxic activity toward gastric epithelial cells [42,43].

#### 1.2.5. Urease Promotes Survival in the Acidic Gastric Environment

A key adaptation of *H. pylori* is the high activity of its urease enzyme, which is composed of two distinct subunits: UreA and UreB [44]. The catalytic activity of urease depends on the presence of nickel ions, the transport and incorporation of which into the active center is controlled by the auxiliary proteins UreE-H [45]. Hydrolysis of urea leads to the formation of ammonia and carbon dioxide. Ammonia binds hydrogen ions, causing a local increase in pH around the bacterial cell and creating conditions that allow *H. pylori* to survive in the stomach. Of particular importance is the membrane protein UreI, which acts as a channel transporting urea into the bacterial cell and is activated under low pH conditions [46]. However, urease also functions as a tissue-damaging agent. Ammonia accumulation can cause mitochondrial dysfunction, epithelial cell damage, and increased production of pro-inflammatory cytokines. In this way, this enzyme simultaneously enables colonization and contributes to the development of chronic gastritis [47,48,49]. 

#### 1.2.6. Cytotoxins as Major Effector Molecules Driving Gastric Epithelial Damage and Chronic Inflammation

VacA (vacuolating cytotoxin A) is one of the main protein toxic agents secreted by *H. pylori*. The expression of *vacA* occurs in virtually all strains of this bacterium, but differences in the regulatory regions and protein structure influence the level of its cytotoxic activity. In line with this, high expression is shown by variants containing the s1 signaling region and the m1 middle region, which are more often associated with a more severe course of infection and a higher risk of pathological changes in the gastric mucosa [50,51,52]. Once released by *H. pylori*, VacA binds to epithelial cell surface receptors and is then internalized, leading to the formation of cytoplasmic vacuoles by disrupting endosomal function and vesicular transport [53,54]. This process includes the modulation of anion channels and proteins regulating cell membrane fusion [55]. Furthermore, VacA affects mitochondrial function, causing loss of membrane potential, increased production of reactive oxygen species, and activation of apoptotic pathways, leading to damage to the epithelial barrier [56,57]. In addition to its direct cytotoxic effects, VacA exhibits immunomodulatory properties by inhibiting the activation and proliferation of T cells. This mechanism limits the effective host immune response and promotes the maintenance of chronic *H. pylori* infection [58,59].

CagA (cytotoxin-associated protein A) is one of the most important virulence factors of *H. pylori*, encoded by *cagA* located within the *cag* pathogenicity island (*cag*-PAI) [60]. The presence of this region enables the expression of the Type IV Secretion System (T4SS), which mediates the translocation of CagA directly into the cytoplasm of host cells. This process requires prior bacterial adhesion, including through the interaction of HopQ with CEACAM receptors on the surface of epithelial cells [61,62,63]. After translocation, CagA is phosphorylated by Src and Abl kinases and then interacts with host signaling proteins, primarily SHP-2 phosphatase. Activation of this pathway leads to dysregulation of epithelial cell proliferation, migration, and polarization, which is associated with the characteristic “hummingbird” phenotype and an increased risk of neoplastic transformation [64,65]. CagA also modulates NF-κB and β-catenin-related pathways, enhancing the production of pro-inflammatory cytokines, including IL-8, and perpetuating chronic inflammation of the gastric mucosa [66]. Strains with more active CagA variants are more often associated with mucosal atrophy, intestinal metaplasia, and the development of gastric cancer [67]. Moreover, the CagA-positive variants are associated with an increased risk of malignant transformation of B-cells (diffuse large B-cell lymphoma), while having no clear effect on the formation of MALT-lymphoma [68].

Despite their different mechanisms of action, VacA and CagA have a complementary effect on the course of infection. VacA limits the immune response and damages epithelial cells, while CagA disrupts signaling pathways regulating host cell function. The joint action of both factors promotes the maintenance of chronic colonization of *H. pylori* and creates conditions for the development of biofilm, which is the next stage of bacterial adaptation and an important factor increasing tolerance to antibiotics.

#### 1.2.7. Biofilm Formation as a Mechanism of *Helicobacter pylori* Persistence and Antimicrobial Resistance

Biofilm formation is one of the key mechanisms enabling *H. pylori* to maintain chronic colonization and increase tolerance to antimicrobial treatment. This process begins with bacterial adhesion to the host cell surface and intercellular aggregation, ultimately leading to the formation of a mature biofilm encased in an extracellular polymeric substance (EPS) composed of polysaccharides, proteins, and extracellular DNA (eDNA) [69]. Numerous *H. pylori* surface proteins, in particular adhesins belonging to the Hop family, are involved in biofilm initiation and stabilization. BabA and SabA are responsible for specific interactions with host cell receptors, while HopQ and AlpA/AlpB are involved in maintaining close contact of bacteria with the epithelial surface and organizing the biofilm structure [36,70]. Biofilm development is also regulated by proteins related to the adaptation of bacteria to the stressful conditions of the stomach environment. Proteins, such as stress response regulators HtrA (high-temperature requirement A), RpoS (RNA polymerase sigma factor S), and SpoT (bifunctional (p)ppGpp synthase/hydrolase), are involved in maintaining cellular homeostasis, responding to oxidative stress, and adapting bacterial metabolism to the limited nutrient availability found in mature biofilms [71,72,73]. NapA (neutrophil-activating protein A) also plays an important role, as it can support bacterial survival in an inflammatory environment by binding iron ions and modulating oxidative stress [74].

Cells in the biofilm exhibit significant metabolic heterogeneity. In addition to actively proliferating bacteria, biofilms contain persister cells, which are characterized by reduced metabolic activity and increased tolerance to antibiotics. Furthermore, the presence of EPS arrays limits the penetration of drugs into deeper layers of the biofilm, reducing their effectiveness against bacteria hidden in the biofilm structure [74,75]. These mechanisms make the elimination of bacteria present in the biofilm much more difficult than in the case of planktonic forms. Biofilm also provides an environment conducive to the genetic adaptation of *H. pylori* [69]. This bacterium has a natural ability to extract foreign DNA from the environment thanks to the ComB/ComFC competence system, which increases the genetic variability of the population and facilitates bacterial adaptation, including the selection of variants with increased antibiotic resistance [76,77]. Apart from the mutation-driven changes in drug resistance of *H. pylori*, biofilm cells of this bacterium are also heavily equipped with efflux pumps, which are membrane proteins associated with the removal of antimicrobial compounds from the interior of bacterial cells [78,79]. As a result, biofilms can facilitate the selection of spontaneous mutations—often acquired via horizontal gene transfer—that lower antibiotic sensitivity, while also triggering processes that passively restrict drug efficacy [62,80]. 

Thus, *H. pylori* biofilm has a dual function: it provides direct protection against unfavorable environmental conditions and creates a niche conducive to the development and consolidation of resistance mechanisms. The complexity of these processes indicates that future therapeutic strategies should include not only the elimination of actively proliferating, planktonic forms of bacterial cells but also the inhibition of biofilm formation and the weakening of virulence mechanisms. 

Despite the availability of effective eradication therapies, antibacterial resistance in *H. pylori* has increased steadily and has become one of the main limitations to successful treatment. Resistance to metronidazole remains the highest among the antibiotics currently used for eradication therapy, exceeding 50% in certain populations, whereas resistance to clarithromycin and levofloxacin has surpassed the 15% threshold—considered critical for the effectiveness of standard triple therapy—in many regions of the world [81,82,83]. In response to this growing problem, the World Health Organization (WHO) has designated clarithromycin-resistant strains as high-priority pathogens for research and development of new therapeutic strategies [84]. Of particular concern is the increasing prevalence of colonization with multidrug-resistant (MDR) *H. pylori* strains, a trend that is significantly driven by repeated eradication failures [81,82]. This phenomenon results in reduced treatment efficacy, the need for subsequent-line therapies, and prolonged persistence of chronic gastric inflammation, creating a significant need for alternative methods of combating infections caused by *H. pylori*.

## 2. Characteristics of Berberine

**Figure 2:**
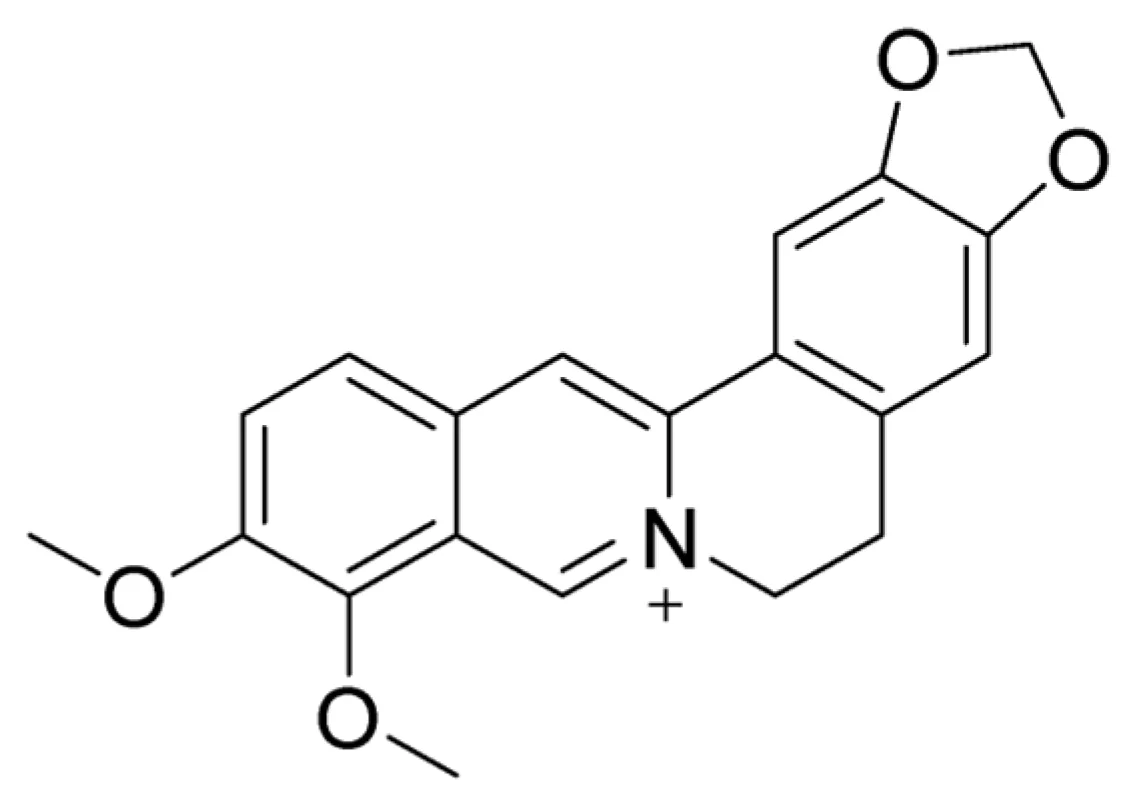
Molecular structure of berberine (BBR).

Given the declining effectiveness of conventional eradication regimens and the increasing prevalence of multidrug-resistant *H. pylori* strains, the development of novel therapeutic strategies has become imperative. In particular, naturally occurring compounds with multifaceted mechanisms of action have attracted considerable attention because of their potential to improve *H. pylori* eradication rates while limiting the development of antimicrobial resistance [85]. In recent years, berberine (BBR; Figure 2), a natural isoquinoline alkaloid found in plants of the genera *Berberis*, *Coptis*, and *Hydrastis*, has emerged as a promising subject of intensive research [86]. 

This alkaloid has aroused considerable interest due to its pleiotropic pharmacological properties and its ability to exert effects at both the cellular and molecular levels. It exhibits a range of biological activities, including antimicrobial [87,88], anti-inflammatory [89,90], antioxidant [91,92], immunomodulatory [93,94], metabolic [95,96], and anticancer [97,98] effects.

The antimicrobial activity of BBR is mediated through multiple mechanisms that target both microbial membrane integrity and key cellular regulatory processes. One of the primary mechanisms is the disruption of bacterial cell membrane integrity, leading to increased membrane permeability, loss of membrane potential, and disturbances in ionic homeostasis [95,99,100]. In addition, BBR disrupts quorum sensing (QS), thereby reducing microbial virulence, activity of efflux pumps, and biofilm formation at multiple stages. All of this ultimately decreases tolerance to external stressors and reverses the multidrug-resistant phenotype [101,102,103,104].

In addition to antimicrobial activity, BBR exhibits potent antioxidant and anti-inflammatory properties through multifaceted regulation of the immune response. One of the best-characterized mechanisms of BBR involves inhibition of the nuclear factor κB (NF-κB) signaling pathway, a central regulator of inflammatory gene expression [105]. Deactivation of NF-κB leads to decreased transcription of pro-inflammatory cytokines, including TNF-α, IL-1β, IL-6, and IL-8, as well as cyclooxygenase-2 (COX-2) and inducible nitric oxide synthase (iNOS) [106,107]. Furthermore, BBR has been shown to inhibit mitogen-activated protein kinase (MAPK) signaling pathways, which further contributes to the attenuation of inflammatory responses [105,108]. At the same time, the BBR-dependent activation of AMP-activated protein kinase (AMPK) leads to the inhibition of pro-inflammatory signaling, improved cellular energy homeostasis, and reduced oxidative stress, which further enhances its anti-inflammatory effect [109].

These properties make BBR a promising compound for further investigation as a novel anti-*H. pylori* agent and a potential component of conventional eradication therapy. Despite promising results from preclinical studies, a comprehensive synthesis integrating the available evidence on mechanisms of action of this alkaloid, its activity against antibiotic-resistant *H. pylori* strains, and its potential role as an element of eradication therapy is still lacking. Therefore, the aim of the present review is to critically summarize the current knowledge regarding the antibacterial properties of BBR against *H. pylori*, with particular emphasis on its activity against MDR strains, mechanisms of action, potential synergistic interaction with eradication drugs, and future clinical perspectives.

## 3. Efficacy of BBR and Its Derivatives Against *Helicobacter pylori* Infection

### 3.1. Indirect Effect on Helicobacter pylori Infection

As outlined in the previous section, chronic inflammation constitutes one of the key mechanisms responsible for the development and progression of gastric diseases associated with *H. pylori* infection. Persistent activation of the immune response results in excessive production of pro-inflammatory mediators, oxidative stress, epithelial damage, and disruption of gastric mucosal integrity, ultimately promoting chronic atrophic gastritis and gastric carcinogenesis. Therefore, apart from direct antibacterial activity, compounds capable of modulating inflammatory pathways may provide additional therapeutic benefits. 

Numerous experimental studies indicate that BBR exerts potent anti-inflammatory effects in the gastrointestinal tract through simultaneous regulation of several signaling pathways involved in the inflammatory response [93,110]. Several in vitro and in vivo studies demonstrated that BBR suppresses NF-κB activation, thereby reducing the production of inflammatory mediators and limiting inflammatory cell infiltration within gastric tissue [105,111,112]. Besides suppressing pro-inflammatory signaling, BBR enhances cellular antioxidant defense by activating the Nrf2/HO-1 pathway. Activation of nuclear factor erythroid 2-related factor 2 (Nrf2) promotes the expression of antioxidant enzymes, including heme oxygenase-1 (HO-1), thereby reducing reactive oxygen species (ROS) accumulation and protecting gastric epithelial cells against oxidative injury [113,114]. Since oxidative stress and inflammation mutually amplify each other during gastric mucosal damage, simultaneous regulation of both processes may substantially improve mucosal healing.

Experimental studies performed in animal models of gastric injury induced by ethanol, non-steroidal anti-inflammatory drugs, or stress further confirmed the gastroprotective properties of BBR [114,115,116]. As demonstrated by different teams, administration of BBR significantly alleviated histopathological damage, reduced neutrophil infiltration, decreased myeloperoxidase activity, and lowered tissue concentrations of pro-inflammatory cytokines [117,118]. These effects were accompanied by preservation of gastric mucosal architecture, restoration of tight junctions, and reduced epithelial apoptosis, suggesting that the anti-inflammatory activity of BBR contributes directly to protection of the gastric barrier.

Delving deeper into the molecular mechanisms of BBR, its influence on *H. pylori*-induced chronic atrophic gastritis should also be considered. Several studies demonstrated that BBR modulates host immune responses rather than acting solely through direct antibacterial mechanisms. Macrophages play a pivotal role in the development of *H. pylori*-associated gastric inflammation, and BBR promotes maintenance of the physiological M1/M2 macrophage balance, thereby limiting excessive production of pro-inflammatory cytokines [119]. BBR also contributed to attenuation of gastritis via silencing BAFF (B-cell-activating factor)-dependent activation of the Th17 response [120]. Additionally, Yang et al. demonstrated that BBR suppresses the IRF8–IFN-γ signaling axis in gastric epithelial cells infected with *H. pylori*, reducing inflammatory activation and attenuating tissue injury [121]. 

**Figure 3:**
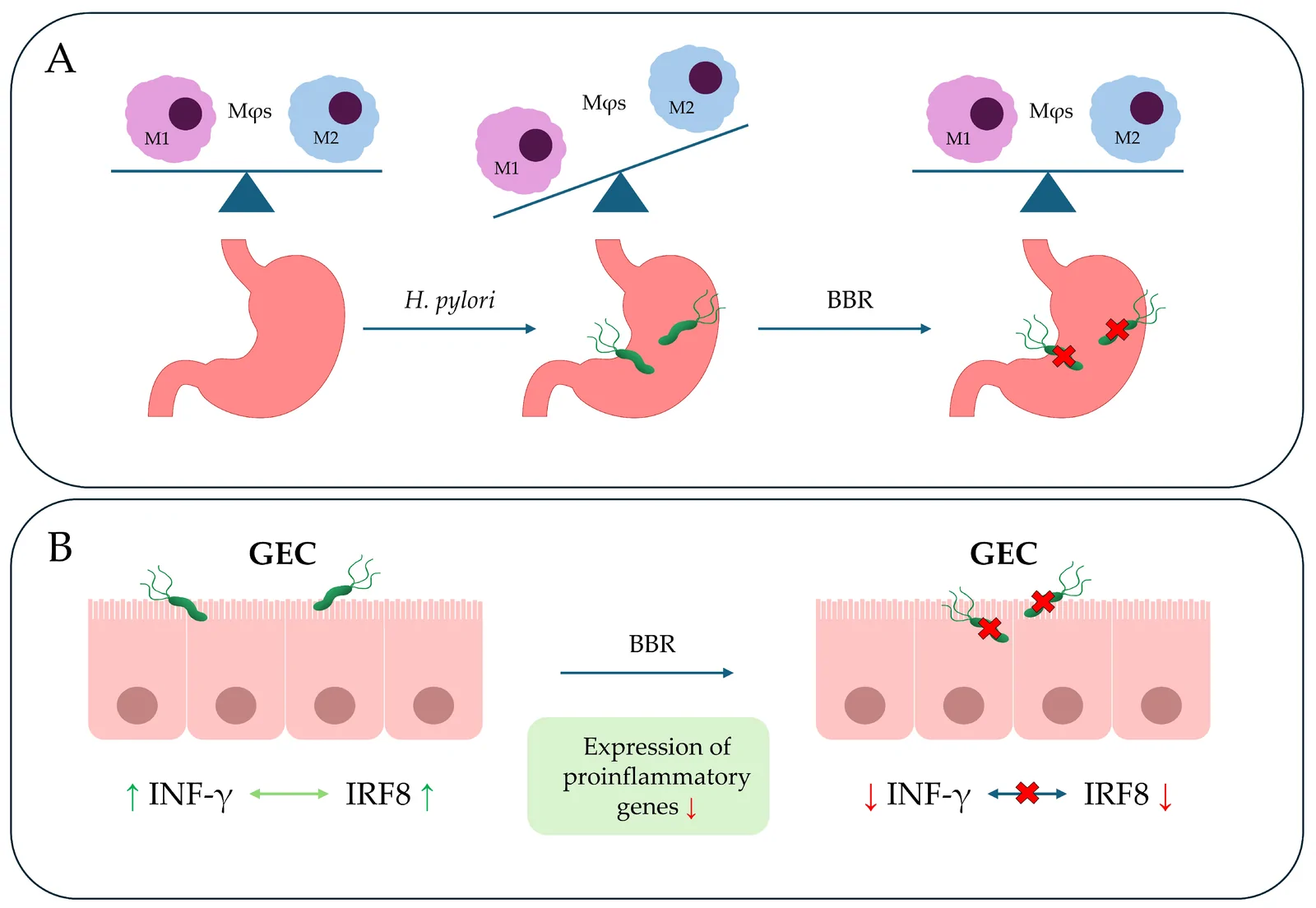
Molecular basis of BBR role in *H. pylori* eradication. (**A**) Berberine restores M1/M2 Mφs homeostasis and eradicates *H. pylori* [119]. (**B**) *H. pylori* stimulates the IRF8-IFN-γ signaling axis, which leads to increased protein expression of IFN-γ, IRF8, Ifit3, IRF1, and Ifit1 genes in GECs; berberine reduces inflammation in GECs through inhibition of the IRF8-IFN-γ pathway [121].

Collectively, these findings suggest that the beneficial effects of BBR in *H. pylori* infection result not only from inhibition of bacterial growth but also from broad modulation of inflammatory and oxidative pathways that contribute to gastric mucosal damage. More details of berberine actions are presented in Figure 3. 

### 3.2. Direct Effect on Helicobacter pylori Infection

#### 3.2.1. In Vitro and Animal In Vivo Research

(a) Anti-virulence Activity

One of the mechanisms underlying the antimicrobial activity of BBR against *H. pylori* is its ability to reduce the expression of virulence factors essential for colonization of the gastric environment and surviving antibiotic treatments. 

Huang et al. conducted a study on the ability of selected plant alkaloids, including BBR, to limit the expression of *hefA*, which encodes the most important and best-studied efflux pump gene of *H. pylori* [122]. Importantly, the combination of BBR with tetracycline or amoxicillin resulted in a reduction in the MIC of these antibiotics similar to that of a routinely applied proton pump inhibitor (PPI). It is worth noting, however, that although Huang et al. investigated the potential interaction between BBR and antibiotics, they did not use the checkerboard assay or determine the fractional inhibitory concentration (FIC) index. Instead, *H. pylori* strains were exposed to BBR or PPIs at one-half of their respective MICs, and the resulting reduction in the MICs of the examined antibiotics was evaluated. For both of them, a two- to fourfold decrease in the MICs of antibiotics was observed in many cases. Although this approach precludes direct comparison with studies using standard synergy assays, the findings suggest an additive interaction, characterized by the ability of a twofold reduction in the MIC of one substance to cause a simultaneous decline in the MIC of the other by a factor of two to several-fold. To gain a deeper understanding of this phenomenon, during molecular analysis, a significant decrease in *hefA* expression after exposure to the alkaloid at subinhibitory concentration was shown. The study demonstrates the possible molecular mechanism by which BBR can restore *H. pylori* sensitivity to one of the key antibiotic groups.

Another important molecular mechanism of BBR activity against *H. pylori* was presented by Tan et al., who detected that epiberberine (a BBR derivative) inhibited bacterial urease by blocking its active site. The findings of this study provide important evidence supporting the potential of this alkaloid, owing to its direct inhibitory effects on pathogen virulence [123]. Similar reports on the ability of *Coptidis chinensis*-extracted BBR to inhibit *H. pylori* urease come from the Li et al. study [124]. Interesting reports on BBR derivative (8-octyl-BBR) activity against *H. pylori* come from the Pan et al. study. In addition to exhibiting greater antibacterial activity than the parent alkaloid, this derivative has been shown to induce multiple molecular alterations at the bacterial cell membrane. It also reduces *H. pylori* virulence by inhibiting urease activity and suppressing the expression of genes encoding flagellar proteins (*flaA* and *flaB*) [125]. As presented above, many studies investigating BBR and its derivatives have focused on their ability to inhibit *H. pylori* urease activity. Although the available findings are promising, current evidence does not permit a definitive ranking of BBR, epiberberine, and 8-octyl-berberine with respect to their anti-urease efficacy. The published research differs substantially in their experimental approaches, ranging from enzyme-based to cell-based assays, making direct comparisons difficult. Nevertheless, among the compounds evaluated, epiberberine is supported by the strongest mechanistic evidence, demonstrating direct interaction with the urease catalytic site.

(b) Antibacterial Activity

A much more extensively studied aspect of BBR activity against *H. pylori* is its ability to directly kill the pathogen.

Several research groups have investigated the anti-*H. pylori* activity of BBR as a constituent of plant extracts. Das et al. evaluated the antimicrobial efficacy of BBR isolated from the methanol extract of the stem of *Berberis aristata* against a double-resistant *H. pylori* isolate. The Kirby-Bauer test showed that 200 µg of BBR produced a zone of inhibition with a diameter of 18 mm [126]. Wu et al. evaluated the activity of BBR as the main component of the extract of *Corydalis yanhusuo* against *H. pylori* and demonstrated the MIC value of 25 µg/mL [127]. Antibacterial properties of the pure BBR and extract from *Berberis vulgaris* were also evaluated against a reference *H. pylori* strain. In this research, the antimicrobial activity was determined using the microdilution method, where BBR exhibited both MIC and MBC of 256 µg/mL [128]. In yet another study, Huang et al. showed the MIC of BBR toward four different MDR *H. pylori* strains (resistant against at least four classically used antibiotics) was in the range of 100–200 µg/mL [122]. The discrepancies in the reported MIC values of BBR most likely reflect methodological and biological differences among studies, including variation in the *H. pylori* strains examined and the susceptibility testing protocols employed. At the same time, the formulation of BBR appears to be the principal determinant. Whereas Huang et al. [122] and Kocić et al. [128] evaluated purified BBR and reported MIC values of 100–256 µg/mL, Wu et al. investigated a BBR-rich plant extract [127]. Although containing a high amount of BBR, the extract contained multiple phytochemicals that may have acted synergistically, potentially accounting for the substantially lower MIC value (25 µg/mL) observed by this team. Overall, the available evidence indicates that native BBR exhibits moderate antibacterial activity against *H. pylori*, with MIC values considerably higher than those of conventional antibiotics. Nevertheless, its retained activity against antibiotic-resistant strains, together with its ability to inhibit efflux pumps at sub-inhibitory concentrations, supports its potential as an adjunct to combination therapies.

On the other hand, other research groups have focused on evaluating the antibacterial activity of various BBR derivatives against *H. pylori*. Guo et al. investigated the properties of a 3,13-disubstituted analog of BBR as a substance with potential antibacterial activity against reference *H. pylori* strains. The compound showed high activity against the tested strains of the pathogen with low MIC values of 0.25–0.5 µg/mL. What is particularly important, is that the BBR derivative also showed activity when checked against clinical MDR strains. Particularly promising was the observed synergistic and additive interaction between the BBR derivative and clarithromycin and amoxicillin, respectively. In addition, an in vivo murine study was conducted, in which various treatment regimens with this BBR derivative were applied. It was reported that both dual therapy (BBR derivative + PPI) and triple therapy (BBR derivative + amoxicillin + PPI) effectively reduced bacterial gastric mucosal load. Electron microscopy highlighted that at the cellular level this treatment led to the perforation of the outer membrane of *H. pylori* isolates. Molecular analysis showed its ability to interfere with key OMPs of *H. pylori* [129]. In another study, significant antibacterial activity of a series of the examined 13-substituted BBR derivatives with a MIC range of 3.125–12.5 µM was demonstrated. The molecular analysis showed increased permeabilization of the bacterial cell membrane after a 2 h exposure to 100 μM of one of the derivatives. What is more, molecular docking found that BBR derivatives lead to inhibition of the FtsZ protein involved in bacterial cell division [130].

Although BBR has attracted considerable attention because of its antimicrobial and gastroprotective properties, its therapeutic application is limited by unfavorable pharmacokinetic characteristics, particularly its extremely low oral bioavailability (absolute systemic absorption from the gastrointestinal tract is in the range of 0.36–0.68%). Following oral administration, BBR is poorly absorbed from the gastrointestinal tract because of its low aqueous solubility and limited intestinal permeability. Moreover, extensive first-pass metabolism in the intestine and liver, together with active P-glycoprotein-mediated efflux, further reduces the amount of unchanged compound reaching the systemic circulation [109,131]. Consequently, plasma concentrations of BBR remain low despite relatively high oral doses. At first glance, these unfavorable pharmacokinetic properties appear inconsistent with the encouraging outcomes reported in several preclinical and clinical studies. However, systemic exposure is unlikely to be the primary determinant of BBR efficacy against *H. pylori*. Unlike drugs that must reach systemic targets, BBR exerts its antibacterial effects predominantly at the mucosal surfaces of the gastrointestinal tract, including the stomach, where *H. pylori* resides. Therefore, high plasma concentrations are not necessarily required to achieve therapeutic activity of BBR. Nevertheless, the poor oral bioavailability of conventional BBR formulations remains a clinically important limitation. The relatively high doses required to achieve therapeutic efficacy, substantial interindividual variability in absorption, and extensive first-pass metabolism may all contribute to inconsistent treatment responses and may partly explain the heterogeneity observed across clinical studies. To overcome these limitations, numerous formulation strategies have been developed to improve the pharmacokinetic profile of BBR. In particular, nanotechnology-based delivery systems—including polymeric nanoparticles, liposomes, solid lipid nanoparticles, nanoemulsions, and micelles—have been shown to enhance its solubility, stability, intestinal absorption, and overall bioavailability [132,133]. Importantly, the rationale for these advanced formulations extends beyond increasing systemic availability of BBR but rather is aimed at improving gastric retention, facilitating penetration through the gastric mucus layer and bacterial biofilm, and increasing the local availability of BBR at the site of infection. These advances may substantially enhance the therapeutic efficacy of BBR and support its future application in the treatment of *H. pylori* infection and other gastric disorders.

To improve the therapeutic applicability of BBR, Cheng et al. evaluated the anti-*H. pylori* activity of BBR encapsulated in mannosylerythritol lipid-B nanomicelles (MEL-B NMs) [6]. This nanocarrier facilitated rapid penetration through the gastric mucus layer and bacterial biofilm while protecting BBR from the acidic gastric environment. As a result, MEL-B NMs and BBR acted synergistically to eradicate *H. pylori*, inhibited biofilm formation, and prevented its further dissemination. In vitro, BBR-loaded MEL-B nanomicelles reduced both biofilm formation and the biomass of established biofilms by approximately 80%. These effects were associated with reduced EPS production and disruption of eDNA packaging within OMVs, leading to impaired *H. pylori* adhesion and biofilm development. The enhanced efficacy of this formulation was further confirmed in a murine model of gastric *H. pylori* infection, where BBR-loaded MEL-B nanomicelles achieved greater reductions in gastric bacterial colonization than standard triple therapy (1.5 × 10^4^ vs. 1.6 × 10^5^ CFU/g of gastric tissue after treatment, corresponding to an approximately one-log (≈90%) additional reduction in bacterial burden). Statistical analysis performed in this study demonstrated that the observed difference between treatment regimens was significant, which is consistent with the biological principle that even moderate reductions in bacterial burden can translate into substantial decreases in virulence and the associated inflammatory response. Although the authors did not directly compare BBR-loaded MEL-B NMs with free BBR under identical experimental conditions, they provided proof-of-concept that this nanoformulation may enhance gastric retention and improve penetration through both the gastric mucus layer and the *H. pylori* biofilm.

In another study, the effect of fucose–chitosan/heparin nanoparticles encapsulated with BBR against *H. pylori* was evaluated both in vitro and in vivo [134]. In an in vitro analysis, a reference *H. pylori* strain was eradicated efficiently at a concentration of 6 µg/mL, while nanoparticles and the alkaloid used alone reduced growth only by 37% and 26%, respectively. With fluorescence microscopy, it was shown that the use of these nanoparticles was associated with significantly higher penetration of BBR into bacterial cells than its free form. An in vivo model proved that exposure to BBR-loaded nanoparticles decreased both bacterial density and urease activity (a *H. pylori* biomarker) in the stomach environment approximately twofold. 

Along similar trends, in another study, the antibacterial properties of nanodrugs developed on the basis of BBR derivatives in combination with rhamnolipids against biofilm and plankton forms of *H. pylori* were investigated [135]. The developed nanodrugs had higher antimicrobial activity than free alkyl BBR derivatives (MIC of 0.78–1.56 µg/mL vs. 1.56–12.5 µg/mL, respectively). Exposure to 100 µg/mL of a BBR-based nanodrug resulted in 90.4% eradication of mature bacterial biofilm, accompanied by significant biofilm disintegration observed by fluorescence microscopy. In vivo studies showed that one alkyl derivative reduced mature *H. pylori* biofilm formation by more than 80%, while the combination of BBR with rhamnolipids enhanced activity through additional disruption of biofilm EPS. Notably, the same derivative inhibited de novo biofilm formation by 99.96% at 6.25 μg/mL, and nanodrug therapy significantly reduced gastric mucosal colonization in a murine *H. pylori* infection model. The near-complete inhibition of *H. pylori* biofilm formation reported by Shen et al. [135] represents one of the most potent anti-biofilm effects described to date for BBR derivatives. Although these findings are highly encouraging, it should be noted that this remarkable activity at micromolar concentrations has not yet been independently replicated. The exceptional efficacy of the tested formulation likely results from the combined effects of the alkylation of BBR, which enhances its antibacterial activity, and the rhamnolipid-based nanoformulation, which facilitates improved penetration through both the gastric mucus layer and the biofilm extracellular matrix. However, because these results are currently supported by a single research group, they should be regarded as promising but preliminary until confirmed by independent studies.

Available evidence indicates that the anti-*H. pylori* activity of BBR and its derivatives is multifaceted, encompassing the inhibition of bacterial adhesion, motility, urease activity, and biofilm formation. At present, however, it is not possible to identify a single dominant molecular target responsible for the greatest reduction in bacterial virulence or the most effective suppression of gastric colonization in vivo. As discussed in the previous sections, successful colonization of the gastric mucosa by *H. pylori* depends on the coordinated action of multiple virulence mechanisms. Therefore, compounds such as BBR and its derivatives, which simultaneously interfere with several of these processes, represent a particularly promising strategy for combating this pathogen. Future studies employing targeted bacterial mutants, protein-specific approaches, or experimental models that selectively disrupt individual virulence mechanisms will be required to define the relative contribution of each anti-virulence effect to the overall inhibition of *H. pylori* colonization. The summary of direct and indirect anti-*H. pylori* activities of BBR and its derivatives is presented in Table 1.

#### 3.2.2. Clinical Trials

Although the literature review reveals very promising results regarding the anti-*H. pylori* activity of BBR in in vitro and in vivo animal models, clinical data provide the final verdict on the substance’s efficacy. 

In an open-label, phase IV randomized trial, the efficacy of 14-day quadruple therapy containing either BBR or a bismuth formulation was compared by a urea breath test. Interestingly, the BBR-based regimen gave non-inferior effects of *H. pylori* eradication in patients relative to the one containing bismuth (intention to treat [ITT] group 90.1% vs. 86.4%, per protocol [PP]: 91.3% vs. 89.6%). Taking into account the frequency of adverse events, no statistically significant difference was found between the two groups (28.6% vs. 35.7%, respectively). In light of such results, the authors postulated that the new form of quadruple therapy can be considered an alternative to the classical bismuth-based [137]. 

In another study, similar results were obtained when comparing a standard 14-day quadruple therapy with the effectiveness of a combination of BBR and standard antimicrobials. In terms of pathogen eradication, both combinations had comparable results (77.5% vs. 76.3% in the ITT group and 85% vs. 81.5% in the PP group). The non-inferior nature of BBR therapy was also demonstrated by a lower frequency of adverse events [138]. 

In a prospective non-inferiority study, Chen et al. evaluated the efficacy of triple therapy containing BBR with amoxicillin and PPI compared to classical quadruple therapy for *H. pylori* eradication. The data obtained were in line with the observations of previous teams, demonstrating BBR-based therapy as noninferior to standard comparators. In terms of eradication, comparable results were recorded in both groups (79.8% vs. 80.9% ITT and 83.6% vs. 85.1% PP) [139]. 

More recently, the efficacy of BBR was evaluated as a component of first-line triple therapy in a randomized clinical trial comparing BBR-based triple therapy with vonoprazan-based and rabeprazole-based quadruple regimens [140]. A total of 300 *H. pylori*-infected patients were enrolled, of whom 263 completed the study. In the ITT analysis, eradication rates were 70.0%, 77.0%, and 69.0% for the BBR-, vonoprazan-, and rabeprazole-treated groups, respectively. The corresponding PP eradication rates were 81.4%, 86.5%, and 78.4%, respectively. No statistically significant differences in eradication efficacy were observed between the treatment groups. Likewise, symptom improvement, treatment compliance, and the incidence of adverse events were comparable across all three regimens. At the same time, it should be noted that the ITT of 70.0% and PP of 81.4% fall below the >90% threshold that many international consensus guidelines consider desirable for an empirical first-line eradication regimen. Although these findings may initially appear discouraging, two important factors should be taken into account. First, the study compared a BBR-based triple therapy with two standard quadruple regimens. The omission of one antimicrobial component in the BBR-based regimen likely reduced the overall synergistic and antibacterial effects of the treatment. Second, the standard quadruple therapies examined in this trial also achieved suboptimal ITT eradication rates (77% and 69%), suggesting that the modest efficacy observed was not specific to the BBR-based regimen, but rather reflected the challenge of eradicating *H. pylori* in a population with a high prevalence of antibiotic-resistant strains.

Taken together, the eradication rates reported for BBR-based therapies vary considerably across clinical trials, ranging from approximately 70% to 90%. This variability is likely multifactorial and should not be attributed solely to the effects of BBR. The available studies differ substantially in their design, with some evaluating BBR as a replacement for a standard component of eradication therapy (e.g., an antibiotic or bismuth salt), whereas others assess its use as an adjunct to conventional treatment. Moreover, the treatment regimens vary with respect to the types and dosages of PPIs and antibiotics administered. Consequently, the available clinical evidence does not allow the independent therapeutic efficacy of BBR to be reliably assessed. Future multicenter trials with standardized BBR formulations, optimized treatment regimens, and antimicrobial susceptibility testing are needed to define the clinical role of BBR in *H. pylori* eradication.

## 4. Conclusions

The increasing failure of *H. pylori* eradication cannot be explained by mutation-driven antibiotic resistance alone. Biofilm formation, bacterial virulence factors, adaptation to the gastric environment, and persistent inflammation collectively create conditions that promote long-term colonization and reduce the effectiveness of conventional therapies. Consequently, therapeutic strategies directed exclusively against bacterial growth are unlikely to fully address the complexity of chronic *H. pylori* infection.

Taking this into context, BBR and its derivatives appear particularly interesting because their activity extend well beyond direct antibacterial effects. The available evidence indicates that these compounds interfere with several mechanisms that determine bacterial persistence, including membrane integrity, urease activity, efflux pump function, bacterial cell division, biofilm development, and the expression of virulence-associated factors. At the same time, they attenuate inflammatory signaling pathways involved in gastric mucosal injury, including NF-κB, MAPK, and IRF8–IFN-γ. This combination of antibacterial, anti-virulence, antibiofilm, and immunomodulatory properties distinguishes BBR and its derivatives from compounds acting through a single molecular target.

Although the published studies differ substantially in experimental design, bacterial strains, investigated compounds, and drug delivery systems, they repeatedly identify the same biological processes as principal targets of BBR-based interventions. Independent investigations consistently report inhibition of urease, disruption of bacterial membranes, impairment of biofilm formation, suppression of efflux pump activity, and improved susceptibility to conventional antibiotics. Collectively, these observations support the biological plausibility of BBR and related compounds as adjuncts to current eradication strategies rather than replacements for antimicrobial therapy. Evidence from randomized clinical trials is also encouraging, demonstrating eradication rates comparable to standard regimens together with good tolerability. Nevertheless, the current clinical data remain limited, and the intrinsically poor oral bioavailability of native BBR continues to represent a major obstacle to its therapeutic application. Recent advances in nanotechnology-based delivery systems are particularly noteworthy because they address precisely those limitations that are most relevant for *H. pylori* infection, namely poor stability in the gastric environment, limited penetration through bacterial biofilms, and insufficient local drug concentrations at the site of infection. Representative nanoformulations, including MEL-B NMs, fucose–chitosan/heparin nanoparticles, and rhamnolipid-based nanodrugs, have demonstrated improved gastric targeting, enhanced antibiofilm activity, and superior antibacterial efficacy compared with native berberine in preclinical studies.

Rather than considering native BBR alone as another naturally derived antibacterial compound, the collective evidence indicates that the broader BBR chemotype—including structurally modified derivatives and advanced formulation strategies—acts as a multitarget resistance-modifying and anti-virulence platform capable of simultaneously interfering with multiple *H. pylori* persistence mechanisms. Future clinical studies should therefore focus on identifying the patient populations most likely to benefit from BBR-based regimens, optimizing formulation strategies, and defining their role in combination with guideline-recommended eradication therapies. Such an approach may prove particularly valuable in regions where multidrug-resistant *H. pylori* strains have become a major cause of treatment failure.

## 5. Materials and Methods

A narrative literature review was conducted to summarize the current evidence regarding the antibacterial activity of BBR and its derivatives against *H. pylori*. The literature search was performed using the PubMed, Scopus, and Web of Science databases. The search was conducted in June and July 2026 and included publications available from database inception until July 2026. Original research articles, clinical studies, systematic reviews, meta-analyses, and narrative reviews published in English were considered.

The search strategy was based on keyword combinations using the Boolean operators AND and OR. The following search terms were applied: "*Helicobacter pylori*", "berberine", "antibacterial activity", "antibiotic resistance", "biofilm", "virulence factors", "urease", "efflux pumps", "nanoparticles", and "eradication therapy". Representative search queries included ("*Helicobacter pylori*" AND "berberine"), ("berberine" AND "antibacterial activity"), ("berberine" AND "antibiotic resistance"), ("berberine" AND "biofilm"), ("berberine" AND "virulence factors"), ("berberine" AND "urease"), ("berberine" AND "efflux pumps"), ("berberine" AND "nanoparticles"), and ("berberine" AND "eradication therapy"), with the Boolean operator OR used where appropriate to include synonymous or closely related terms and broaden the search.

The retrieved publications were screened for relevance based on their titles, abstracts, and full texts. Particular emphasis was placed on studies investigating the molecular mechanisms of berberine activity, its effects on antibiotic-resistant *H. pylori* strains, synergistic interactions with conventional eradication therapies, nanoformulation-based drug delivery systems, and available clinical evidence. Additional relevant publications were identified through manual screening of the reference lists of selected articles. A total of 140 articles were included in the final narrative synthesis.

## Figures and Tables

**Table 1:** Molecular mechanisms underlying the direct and indirect anti-*H. pylori* activity of BBR and its derivatives.

Target	Tested Compound	Molecular Effect	Impact on *H. pylori*	Experimental Model	References
**DIRECT**					
**Bacterial cell** **membrane**	8-octyl-BBR; 13-substituted BBR derivative	Disrupts membrane integrity Damages to outer cell membrane	Loss of membrane integrity Leakage of intracellular components Bacterial death	In vitro and animal model	[129,130]
**Divisome**	13-substituted BBR derivatives	Inhibits FtsZ involved in bacterial cell division	Arrest of bacterial cell division and growth	In vitro and molecular docking	[130]
**Flagellar apparatus**	8-octyl-BBR	Downregulates flagellar protein genes (*flaA*, *flaB*)	Reduced bacterial motility and colonization capacity	In vitro	[125]
**Outer membrane** **proteins (OMPs)**	8-octyl-BBR	Alters the expression/function of outer membrane proteins	Reduced bacterial virulence and impaired membrane integrity	In vitro and molecular docking	[129]
**Efflux pumps**	Native BBR	Downregulates *hefA* expression	Restores susceptibility to antibiotics and attenuates MDR	In vitro	[122]
**Urease**	Epiberberine; BBR from *Coptidis chinensis*; BBR-loaded fucose–chitosan/heparin nanoparticles	Decreases the urease activity	Impaired survival in acidic gastric environment and reduced colonization	In vitro, molecular docking, and animal model	[123,124,134]
**eDNA-containing OMVs**	BBR-loaded MEL-B nanomicelles	Disrupts eDNA packaging within OMVs	Reduced bacterial adhesion and impaired biofilm development	In vitro and animal model	[136]
**Biofilm formation**	BBR-loaded MEL-B nanomicelles; alkyl BBR derivatives with rhamnolipid nanodrugs	Inhibits initial adhesion, biofilm maturation, and EPS	Reduced biofilm biomass and increased susceptibility to eradication therapy	In vitro and animal model	[135,136]
**INDIRECT**					
**Host inflammatory signaling**	Native BBR	Inhibits NF-κB, MAPK, and IRF8–IFN-γ signaling pathways	Reduced gastric inflammation and tissue injury; indirect suppression of *H. pylori* persistence	In vitro and animal model	[119,121]
**Macrophage** **polarization**	Native BBR	Restores physiological M1/M2 macrophage balance	Reduced chronic inflammatory response associated with *H. pylori* infection	Animal model	[119]

## Data Availability

No new data were created or analyzed in this study. Data sharing is not applicable to this article.

## Abbreviations

The following abbreviations are used in this manuscript:

Abl	tyrosine-protein kinase Abl
AMPK	AMP-activated protein kinase
BabA	blood group antigen-binding adhesin
BAFF	B-cell-activating factor
BBR	berberine
β-Cat	beta-catenin
CagA	cytotoxin-associated protein A
CEACAM	carcinoembryonic antigen-related cell adhesion molecule
Cl^−^	chloride anion
CO_2_	carbon dioxide
COX-2	cyclooxygenase-2
CytC	cytochrome C
DLBCL	diffuse large B-cell lymphoma
eDNA	extracellular DNA
EPS	extracellular polymeric substances
*flaA*	flagellin A gene
*flaB*	flagellin B gene
FIC	fractional inhibitory concentration
FtsZ	filamenting temperature-sensitive protein Z
GEC	gastric epithelial cells
GECs	gastric epithelial cells
H^+^	proton
H_2_O	water
*hefA*	*Helicobacter* efflux pump A gene
HO-1	heme oxygenase-1
HtrA	high-temperature requirement A protein
IFN-γ	interferon-gamma
IL-1β	interleukin-1 beta
IL-6	interleukin-6
IL-8	interleukin-8
iNOS	inducible nitric oxide synthase
IRF8	interferon regulatory factor 8
ITT	intention-to-treat
MALT	mucosa-associated lymphoid tissue
MAPK	mitogen-activated protein kinase
MBC	minimum bactericidal concentration
MCPs	methyl-accepting chemotaxis proteins
MDR	multidrug-resistant
MEL-B	mannosylerythritol lipid-B
Mφs	macrophages
MIC	minimum inhibitory concentration
NF-κB	nuclear factor kappa B
NH_3_	ammonia
NH_4_^+^	ammonium cation
NMs	nanomicelles
Nrf2	nuclear factor erythroid 2-related factor 2
OMPs	outer membrane proteins
OMVs	outer membrane vesicles
P	phosphorylated
PLA_2_	phospholipase A_2_
PMF	proton motive force
PP	per protocol
PPI	proton pump inhibitor
QS	quorum sensing
RAS	Rat Sarcoma Virus
ROS	reactive oxygen species
SabA	sialic acid-binding adhesin
SHP-2	Src homology 2 domain-containing protein tyrosine phosphatase 2
Src	proto-oncogene tyrosine-protein kinase Src
T4SS	type IV secretion system
Th17	T helper 17
TJs	tight junction proteins
TLR2	Toll-like receptor 2
TNF-α	tumor necrosis factor-alpha
VacA	vacuolating cytotoxin A
WHO	World Health Organization.
